# A Review of Postmortem Findings in Patients With COVID-19

**DOI:** 10.7759/cureus.9438

**Published:** 2020-07-28

**Authors:** Adenike O Eketunde, Sai Priyanka Mellacheruvu, Philip Oreoluwa

**Affiliations:** 1 Public Health, University of Massachusetts Lowell, Lowell, USA; 2 Public Health, John Hopkins School of Public Health, Baltimore, USA

**Keywords:** postmortem pathological findings, postmortem, covid19, lungs, cardiovascular, hematology, renal

## Abstract

Multiple public health problems have been caused by various coronavirus strains over the last few years, such as the middle eastern respiratory syndrome (MERS), severe acute respiratory syndrome (SARS), and COVID-19. COVID-19, which is also known as coronavirus disease 2019, was first detected in Wuhan, China, and has significantly impacted people's health and lives. Additionally, it has led to a pandemic, and the virus has spread to over 121 countries worldwide. There is numerous information available regarding this virus. A detailed and extensive study of the morphological and histopathological findings will help understand and diagnose the disease. As it is a new disease, it is challenging to understand the mechanism of the action and disease pathology due to the limited availability of data from autopsies or biopsies. However, as the detailed mechanism of injury remains unclear, this paper aims to review the postmortem gross and histopathological findings of various organs that have been affected with coronavirus, focusing on the pulmonary, cardiac, and hematologic findings. This paper emphasizes the postmortem findings of the effect of the coronavirus disease on multiple organ systems.

Advance search of the keywords on PubMed was used, limiting the search to the last five years. The eligible article is narrowed based on relevance containing postmortem findings of the novel virus; COVID-19. A total of 25 full-text articles were selected and used in the review of this paper.

## Introduction and background

COVID-19 is a pandemic that has affected a large percentage of the world’s population. The total number of COVID-19 cases worldwide as of July 17, 2020, is over 13.9 million, which has led to over 593,000 deaths [1]. These numbers are still rising. The severe acute respiratory syndrome coronavirus 2 (SARS-CoV-2) virus originated in Wuhan, China, and has spread rapidly across the world. People around the globe are following strict measures to contain the virus, and there is ongoing research into treatment for this fatal virus. The respiratory and immune systems are most affected by coronavirus strains.

COVID-19 is a novel coronavirus that has ribonucleic acid (RNA) as nuclear material. It was declared a pandemic by the World Health Organization on March 11, 2020 [2]. The SARS-COV-2 virus has a similar genomic sequence, clinical manifestations, and biological behavior to SARS-COV [3]. The four major structural proteins are as follows: the spike surface glycoprotein, small envelope protein, matrix protein, and nucleocapsid protein. The spike protein binds to the host receptors via the receptor-binding domains of the angiotensin-converting enzyme 2 (ACE2) [4]. The ACE2 protein is also present in various human organs, including the respiratory system, gastrointestinal tract, liver, kidney, spleen, lymph nodes, bone marrow, and brain [3]. SARS-COV-2 is believed to be more virulent than SARS-COV, as the virus affects various organ systems in the human body [3]. Our study provides a review of the postmortem findings of the above-mentioned organs (see the Appendix), which will significantly improve the current understanding of the disease.

There is a wide range of clinical spectrums of COVID-19, which ranges from the asymptomatic or paucisymptomatic forms to the conditions that are characterized by respiratory failure, which requires mechanical ventilation and admission to the intensive care unit. Very severe cases can lead to multi-organ dysfunction syndrome (MODS), which can result in sepsis or shock. The clinical manifestations have been divided into the following three categories based on the severity:

Mild disease

This is an uncomplicated or mild illness. Individuals show mild symptoms, such as a mild fever, dry cough, sore throat, nasal congestion, malaise, headache, or myalgias. This comprises 81% of the total cases, while some patients with moderate disease present with respiratory symptoms such as cough, shortness of breath, and tachypnea. No signs and symptoms of severe disease are present [5].

Severe disease

This occurs in 14% of cases. The symptoms in these patients range from dyspnea, a respiratory rate of >30, and a blood oxygen saturation of less than 93% [5]. They present with severe pneumonia, acute respiratory distress syndrome, or sepsis. Even in severe forms of disease fever can be moderate or absent [5].

Critical

These patients present with respiratory failure, cardiac injury, septic shock, and/or MODS, or multi-organ failure (MOF). This comprises 5% of the total cases [5].

The individuals who develop symptoms and/or are exposed to the virus are tested for COVID-19. A nasal swab is collected as a sample. During the analytic stage, real-time reverse transcription polymerase chain reaction (RT-PCR) is the molecular test of choice for the diagnosis of SARS-Cov-2 infections, whereas antibody-based tests were introduced as a supplemental tool. Post-analytically, the results should be interpreted carefully with the help of both the molecular and serological findings [3].

People across the world are practicing social and physical distancing to control the spread of this fatal virus. Those who have been exposed to someone who is infected with the disease must be quarantined, and those who test positive and develop symptoms must isolate for 14 days. According to a study conducted in China, the median incubation period for COVID-19 was estimated to be 5.1 days with a 95% confidence interval with a range of 4.5 to 5.8 days. Approximately 97.5% of those who will develop symptoms will do so within 11.5 days with 95% CI, with a range of 8.2 to 15.6 days [6].

## Review

The causes of death from COVID-19 range from acute respiratory distress syndrome (113; 100%), type I respiratory failure (18/35; 51%), sepsis (113; 100%), acute cardiac injury (72/94; 77%), heart failure (41/83; 49%), alkalosis (14/35; 40%), hyperkalemia (42; 37%), acute kidney injury (28; 25%), and hypoxic encephalopathy (23; 20%) [7].

Several studies have demonstrated how the COVID-19 hyperinflammatory response is one of the primary causes of death that affects the heart vessels, liver, kidney, and other organs. Macrophages are the major cells that are activated in response to an injury by supporting and activating the stem/progenitor cells, clearing the damaged tissue, remodeling the extracellular matrix to prepare the scaffolding for regeneration, and promoting angiogenesis [8-10].

Postmortem pulmonary findings in COVID-19 patients

The bronchoalveolar fluid in patients with severe COVID-19 has shown a large quantity of chemokine from the macrophages; this was also evident in the immunostaining of postmortem tissue from a COVID-19 patient. The CD169+ lymph node, subcapsular, and splenic marginal zone macrophages expressed the SARS-CoV-2 entry receptor ACE2. They showed that these macrophages contained the SARS-CoV-2 nucleoprotein (NP), although the small conditional RNA (scRNA)-seq analysis of human tissue failed to identify an ACE2 expression on most tissue-resident macrophages [11]. According to Merad and Martin's study, the hyper inflammation in severe COVID-19 patients shared similarities with cytokine release syndromes, including macrophages activation syndrome. This leads to an increase in the production of cytokines, such as interleukin (IL)-6, IL-7 and tumor necrosis factor (TNF), as well as inflammatory chemokines, including CC-chemokine ligand 2 (CCL2), CCL3 and CXC-chemokine ligand 10 (CXCL10), and the soluble form of the α-chain of the IL-2 receptor. This then leads to the hypothesis of the dysregulated activation of the mononuclear phagocyte (MNP) compartment and contributes to the COVID-19-associated hyperinflammation [9].

Minimally invasive autopsies of three COVID-19 patients in Chongqing, China revealed damage to the alveolar structure with minor serous and fibrin exudation and hyaline membrane formation [8]. The cells that were identified in the alveolar structure were mainly macrophages and monocytes. The other cells that were identified included multinucleated giant cells, lymphocytes, eosinophils, and neutrophils. The blood vessels of the alveolar septum were congested and edematous with monocyte and lymphocyte infiltration, mainly macrophages and monocytes [8]. The findings show that the coronavirus particles in the bronchial epithelia and type II alveolar epithelia and immunohistochemical staining were positive for the 2019-nCoV antigen, and the PCR analysis was positive for 2019-nCoV nucleic acid. The findings from the other organs showed degeneration and necrosis of the parenchyma cells and hyaline thrombus formation in the small blood vessels; however, there was no evidence of coronavirus infection in the cells [8]. COVID-19 has a 58% incidence of deep venous thrombosis, of which 33% of patients had pulmonary embolism as a direct cause of death [12]. Another autopsy of an 84-year-old woman in China who died of COVID-19, which took place five hours after her death, showed diffuse alveolar damage with a prominent hyaline membrane and inflammatory cells with prominent plasma cells in the alveolar septa. Other observations included an interalveolar hemorrhage, vascular congestion, hyperplasia of type 2 pneumocytes, and phagocytosis in the lungs, spleen, and lymph nodes. The glomeruli of both kidneys were marked by microthrombi, which suggests early signs of disseminated intravascular coagulation [13].

Postmortem cardiovascular findings in COVID-19 patients

Like other viruses, coronavirus is associated with cardiomyopathy and myocarditis. Infected patients may develop lymphocytic, eosinophilic, or giant cell/granulomatous myocardial inflammation, which results in infectious dilated cardiomyopathy [14]. The myocardial damage from COVID-19 that led to the death is about 7% and contributed to death in 33% of patients [15]. An autopsy of a patient that died of COVID-19 showed pericardial effusion. The macroscopic finding was described as "gray, red, fish-like," and microscopically showed diffuse inflammatory infiltrates that were composed of lymphocytes and macrophages with prominent eosinophils, which confirmed inflammation. The findings showed primary inflammation of the interstitium, which is associated with multiple foci of myocyte necrosis. It was present in both the right and left ventricles without vascular inflammation or fibrinoid necrosis, which was not angiocentric, granulomatous, or associated with vasculitis [16]. The gross findings from the heart of a COVID-19 patient showed cardiomegaly with right and left ventricular dilatation. The microscopy showed scattered individual cell myocyte necrosis and did not identify a significant lymphocytic inflammatory infiltrate, which is suggestive of viral myocarditis. This is a mechanism that is described by Chen et al. and states that the pericytes might be infected with SARS-CoV-2 and cause capillary endothelial cell or microvascular dysfunction, which could cause individual cell necrosis [17,18].

Hematology, renal and other findings in COVID-19 patients

A study that was performed in Brazil showed fibrin thrombi in the alveolar arterioles of 80% of patients with COVID-19. This is evidence of the hypercoagulable state in severely ill patients, which leads to a ventilation-perfusion mismatch in the lungs and a peripheral ischemic event [13]. The hypercoagulable state has been linked to a poor prognosis in patients with severe COVID-19, which leads to a microthrombi formation in the lungs, lower limbs, hands, brain, heart, liver, and kidneys, as a result of the activation of the coagulation pathway. This is mediated by the inflammatory cytokine, which can be explained by the hyperinflammation that is caused by COVID-19, as mentioned above [18-19]. The hypercoagulable states can also explain the other findings, including those from the brain. A neuropathological finding from a patient who died of COVID-19 complications revealed features of vascular and demyelinating etiologies. The neuropathological findings in this patient showed hemorrhagic white matter lesions throughout the cerebral hemisphere with a surrounding axonal injury, as well as macrophages. The subcortical white matter of the brain had clusters of macrophages, a range of associated axonal injuries, a perivascular acute disseminated encephalomyelitis (ADEM)-like appearance, and focal microscopic areas of necrosis with a central loss of white matter and marked axonal injury [20].

Autopsy reports of 10 patients from Hospital Graz II, Austria, which is the second-largest public and academic teaching hospital in Austria, showed thrombosis of the small-sized and mid-sized pulmonary artery at different stages in all patients, bronchopneumonia in six patients, and hepatic congestion in eight patients. The other findings in the liver included hepatic steatosis, portal fibrosis, lymphocytic infiltrates and ductular proliferation, lobular cholestasis, and acute liver cell necrosis, along with central vein thrombosis. Other common findings included renal proximal tubular injury, focal pancreatitis, adrenocortical hyperplasia, and lymphocyte depletion of the spleen and lymph nodes [21]. Although it was not a common feature, there were also findings of direct renal infection with the presence of viral particles that were morphologically identical to SARS-CoV-2. Additionally, the identification of the tubular isometric vacuolization using light microscopy correlated with the double-membrane vesicles that contained vacuoles, which were observed using electronic microscopy [22]. In another autopsy that was performed by the University Medical Center Hamburg-Eppendorf on 12 patients, four deaths were identified as a direct cause of a pulmonary embolism, which arose from the deep veins of the lower extremities. The autopsy revealed deep vein thrombosis in seven out of 12 patients, where thromboembolism had not been suspected at the time of death [12]. The kidney can also be damaged with marked microthrombi of the glomerulus, which can be a sign of disseminated intravascular coagulation [23].

The hematologic dysfunction can be explained by the virus-induced procoagulant and coagulopathy, virus invasion, and damage of the lymphocytes. This resulted in lymphopenia, which is the depletion of white pulp that leads to a predisposition to deep vein thrombosis and pulmonary thromboembolism [24].

Extrapulmonary findings from certain comorbidities, such as diabetes and hypertension, are related to septic shock, and other changes, including superficial perivascular dermatitis, orchitis, myositis, myocarditis, alteration in the renal glomeruli, and endothelium of small vessels and cerebral cortex, are related to the COVID-19 virus [20].

## Conclusions

The most common postmortem findings were the pulmonary findings, which included diffuse alveolar damage in almost all cases. Other pulmonary findings, including acute respiratory distress syndrome and tissue from the lungs, were usually positive for the 2019-nCoV antigen. The other significant extrapulmonary findings require further research, as understanding the pathological findings will be helpful for the treatment plans, including the use of anticoagulants in patients in hypercoagulable states. There is a strong association with the hyperinflammatory state, which can be explained by most of the signs and symptoms that are exhibited by COVID-19 patients, including most of the pathological findings.

## Appendices

**Table 1:** Postmortem Findings in Patients with COVID19

Lungs/Pulmonary vascular	Cardiovascular
Macroscopic findings: Widespread thrombosis in small and medium pulmonary arteries	Macroscopic findings: Cardiomyopathy
Evidence of Bronchopneumonia	Myocarditis
Pulmonary embolism	Pericardial effusion
Microscopic findings: Diffuse alveolar damage with prominent hyaline membrane and inflammatory cells with prominent hyaline membrane and inflammatory cells with prominent plasma cells in alveolar septa	Microscopic findings: Lymphocytic eosinophilic or giant cell/granulomatosis. Myocardial inflammation which results in dilated cardiomyopathy
Hyperplasia of Type 2 pneumocyte cells	

**Table 2:** Postmortem Findings in Patients with COVID19

Liver/Hepatic cells	Brain and Other Neurological Systems	Kidneys	Pancreas	Adrenal Glands
Macroscopic findings: Hepatic steatosis	Macroscopic findings: Hemorrhagic white matter lesions throughout the cerebral hemisphere	Renal proximal tubular injury	Focal Pancreatitis	Adrenocortical hyperplasia
Portal fibrosis Lobular cholestasis Acute Liver necrosis Central vein thrombosis		Wide spread glomeruli microthrombi		
Microscopic findings: Lymphocytic infiltrate and ductal proliferation	Microscopic findings: Subcortical white matter of the brain had clusters of macrophages, a range of associated axonal injuries, a perivascular acute disseminated encephalomyelitis (ADEM) like appearance and focal microscopic areas of necrosis and marked axonal injury.			
	Axonal injuries as well as macrophages			
